# Health care expenditure for hospital-based delivery care in Lao PDR

**DOI:** 10.1186/1756-0500-5-30

**Published:** 2012-01-14

**Authors:** Daovieng Douangvichit, Tippawan Liabsuetrakul, Edward McNeil

**Affiliations:** 1 National Institute of Public Health, Ministry of Health, Vientiane capital, Lao PDR; 2 Epidemiology Unit, Faculty of Medicine, Prince of Songkla University, Hat Yai, Songkhla 90112, Thailand

## Abstract

**Background:**

Delivery by a skilled birth attendant (SBA) in a hospital is advocated to improve maternal health; however, hospital expenses for delivery care services are a concern for women and their families, particularly for women who pay out-of-pocket. Although health insurance is now implemented in Lao PDR, it is not universal throughout the country. The objectives of this study are to estimate the total health care expenses for vaginal delivery and caesarean section, to determine the association between health insurance and family income with health care expenditure and assess the effect of health insurance from the perspectives of the women and the skilled birth attendants (SBAs) in Lao PDR.

**Methods:**

A cross-sectional study was carried out in two provincial hospitals in Lao PDR, from June to October 2010. Face to face interviews of 581 women who gave birth in hospital and 27 SBAs was carried out. Both medical and non-medical expenses were considered. A linear regression model was used to assess influencing factors on health care expenditure and trends of medical and non-medical expenditure by monthly family income stratified by mode of delivery were assessed.

**Results:**

Of 581 women, 25% had health care insurance. Health care expenses for delivery care services were significantly higher for caesarean section (270 USD) than for vaginal delivery (59 USD). After adjusting for the effect of hospital, family income was significantly associated with all types of expenditure in caesarean section, while it was associated with non-medical and total expenditures in vaginal delivery. Both delivering women and health providers thought that health insurance increased the utilisation of delivery care.

**Conclusions:**

Substantially higher delivery care expenses were incurred for caesarean section compared to vaginal delivery. Three-fourths of the women who were not insured needed to be responsible for their own health care payment. Women who had higher family incomes were able to pay for more non-medical care expenses. The effect of health insurance on service utilization was noted by women and SBAs. To achieve the goal of utilizing delivery care in the hospitals, coverage of health insurance needs to be expanded.

## Background

In 2008, 358,000 maternal deaths were reported globally, of which 99% occurred in developing countries [1]. Fifteen percent of pregnant women have an increased risk, which is unpredictable, of obstetric complications and death based on World Health Organization (WHO) estimates. As a result, delivery by a skilled birth attendant (SBA) in hospital is recommended in order to provide prompt treatment and thus reduce the mortality rate of mothers and their newborn [1,2]. However, high health care expenses impede the utilization of hospital-based care, especially in countries where the majority of people are forced to pay out-of-pocket [3,4]. Evidence has shown that a high rate of out-of-pocket expenses occurs in countries with a high rate of home deliveries [4]. Out-of-pocket expenses for health care have been shown to be associated with an increased risk of impoverishment [5,6] and catastrophes, particularly in low-income countries [3].

Health care expenses vary depending on the type of services provided [6]. For delivery care, expenses incurred for vaginal delivery is much less than for caesarean section [7-9]. To reduce the risk of catastrophic impoverishment for health care utilization, health insurance was initiated in various countries [10-12]. Availability of health insurance increased health care utilization in Senegal [13] and prenatal care and birth delivery assistance as part of maternal health care in Turkey [11]. However, reimbursement of health insurance included only the costs for medical care.

From World Health Statistics, the maternal mortality ratio in Lao People's Democratic Republic (Lao PDR) in 2005 was 405 per 100,000 live births and the proportion of births in facilities was 18.5%, while in 2006 half of all health expenditures was paid out-of-pocket [14]. The national health insurance program consists of mandatory and voluntary systems. The Civil Servant Scheme (CSS) for government employees and the Social Security Organization (SSO) for employees of private companies are the mandatory systems of which the beneficiaries are managed by the Ministry of Labour Social Welfare (MoLSW). Community-based Health Insurance (CBHI) for the general population and Health Equity Funds (HEF) for the poor are voluntary systems managed by the Ministry of Health [15]. However, implementation of all health insurance schemes is not universal throughout the country [16].

Health care expenditure includes both medical and non-medical expenses and both should be considered when assessing health economic aspects [7,17]. Few studies have assessed both medical and non-medical care expenditure when assessing costs of delivery care and the effect of health insurance on hospital-based delivery care utilization [10-12]. This study, therefore, aimed to estimate the total health care expenditure for hospital-based delivery care among women giving birth by vaginal delivery and those who had caesarean section, to determine the association between availability of health insurance and monthly family income with health care expenditure and to assess the effect of health insurance for hospital-based delivery care from the perspectives of the women and the skilled birth attendants in Lao PDR.

## Methods

A cross-sectional study was carried out from June to October 2010 in Bolikhamxay and Khammouane Provincial Hospitals in Lao PDR where comprehensive emergency departments are available. Both hospitals are a 150-bed facility. In 2009, Bolikhamxay hospital had 845 deliveries while Khammouane hospital had 1250.

This study protocol was approved by the National Ethics Committee for Health Research in Lao PDR and the Institute Ethical Research Committee of the Faculty of Medicine, Prince of Songkla University, Thailand.

All pregnant women who delivered at the two hospitals were eligible for inclusion in the study. Due to the lack of available information concerning variation in medical and non-medical health care expenses, a pilot study was conducted involving 60 women who delivered in the participating study hospitals. The standard deviation of health care expenses for delivery care services was 76 USD and this was used to calculate the sample size for the main study. To compensate for 20% of additional variation, at least 545 women were needed to estimate health care expenses with a precision of 7 USD and a 95% level of confidence. All skilled birth attendants (SBAs) who were working during the study period were also included in the study.

Permission from the directors of both hospitals was approved before interviews were obtained. In each hospital, one person, who was not a health provider nor affiliated with the hospital, was trained on how to conduct the interviews. Other information in this study was obtained from the hospital medical records and the interviews from the women and/or their family members.

All eligible women were approached by the trained interviewer on the day of discharge. After the women gave written consent, interviews were carried out in a private room to determine non-medical care expenses incurred. Medical care expenses were reviewed from medical records and receipts of payment. In addition, both women and SBAs were asked for their perception on the effect of available health insurance on the quality of delivery services. All women and SBAs were informed that their identity would be kept anonymous and responses kept confidential. All interviews were administered on the day of discharge to ensure that all expenditures were recorded.

Medical care expenses included any expenditure due to medication, operation, laboratory investigation, hospital stay and commodities used in the hospital. Non-medical care expenses included staff gratuities, other drugs bought outside the hospital, total transportation costs from home to hospital, food consumed (hospitals in Lao PDR do not provide food to their patients) and productivity loss of companions during the woman's hospital stay. Total health care expenditure was calculated by combining the medical and non-medical care expenses.

Information obtained from the interviews of the women included demographic and socio-economic data, type of health insurance and obstetric characteristics. Demographic and socio-economic data included age, religion, ethnicity, occupation, education and monthly family income. Obstetric characteristics included mode of delivery, gravidity, parity, and complications associated with the current pregnancy and delivery. The coverage and type of health insurance were also recorded.

The effect of health insurance on delivery care services was collected by interviewing the pregnant women and their SBAs. For pregnant women who were uninsured the following three questions were asked: "*Do you know that health insurance is available?", "Do you know that you have a right to have health insurance?" *and *"If you had health insurance, would it influence how you accessed a hospital?"*. For women who had health insurance, the following question was asked: *"Is the health insurance you have important for you in making the decision to deliver in the hospital?"*. SBAs were asked the following two questions: "*Do you think delivery in a hospital would increase if every pregnant woman had health insurance?" *and *"Do you think the health insurance system has an effect on the quality of service?"*.

The data were analysed using R software version 2.11.0 (The R Foundation for statistical computing, Austria. 2010). Continuous variables were checked for normality using the Shapiro-Wilk test. Descriptive statistics were presented as means and medians for normally and non-normally distributed variables, respectively.

Women's characteristics and health care expenses were compared for the two modes of delivery using the chi-square test, rank sum test or t-test as appropriate. Factors associated with expenditure were identified by a linear regression model using a backward stepwise technique. A *p*-value of less than 0.05 was considered significant. Linear regression was used to assess the trend of expenditure and monthly family income by mode of delivery.

## Results

### Health care expenditure

Of 581 women enrolled in the study, 514 had vaginal delivery and 67 delivered by caesarean section. The age of the women ranged from 16 to 46 years (mean ± SD: 25.2 ± 5.3 years). Women's characteristics by mode of delivery are shown in Table 1. Demographic and socio-economic characteristics were not significantly different between modes of delivery. A significantly higher proportion of pregnancy and delivery complications were found in women having caesarean section compared to vaginal delivery.

**Table 1:** Comparison of women's characteristics by mode of delivery

	Mode of delivery		*P *value
		
	Vaginal delivery n (%)	Caesarean section n (%)	
**Demographic and socio-economic characteristics**			

Hospital			0.07
A	234 (45.5)	39 (58.2)	
B	280 (54.5)	28 (41.8)	
Age (years)			0.23
< 20	68 (13.2)	4 (6.0)	
20-34	408 (79.4)	58 (86.6)	
≥ 35	38 (7.4)	5 (7.4)	
Religion			1.00
Buddhist	489 (95.1)	65 (97.0)	
Christian	7 (1.4)	0	
Animist	18 (3.5)	2 (3.0)	
Ethnicity			0.56
Laolum	487 (94.7)	65 (97.0)	
Other	27 (5.3)	2 (3.0)	
Education			0.21
Primary school or lower	198 (38.5)	20 (29.9)	
Secondary school or higher	316 (61.5)	47 (70.1)	
Occupation			0.28
Housewife	175 (34.1)	18 (26.9)	
Government officer	87 (16.9)	15 (23.9)	
Merchant	89 (17.3)	16 (22.4)	
Farmer	163 (31.7)	18 (26.8)	
Husband's occupation			0.08
Government officer	181 (35.2)	33 (49.3)	
Merchant	35 (6.8)	3 (4.5)	
Farmer	298 (58.0)	31 (46.3)	
Monthly family income (USD)			0.11
Median (IQR)	97.5 (50,145)	120 (57.5,200)	

**Obstetric characteristics**			

Gravidity			0.04
Primigravida	258 (50.2)	43 (64.2)	
Multigravida	256 (49.8)	24 (35.8)	
Parity			0.11
0	258 (50.2)	44 (65.7)	
1-2	206 (40.1)	20 (29.8)	
3-4	40 (7.8)	2 (3.0)	
> 4	10 (1.9)	1 (1.5)	
Pregnancy complications			< 0.001
No	466 (90.7)	51 (76.1)	
Yes	48 (9.3)	16 (23.9)	
Delivery complications			0.01
No	507 (98.6)	62 (92.5)	
Yes	7 (1.4)	5 (7.5)	

IQR: Inter-quartile range

**Figure 1:**
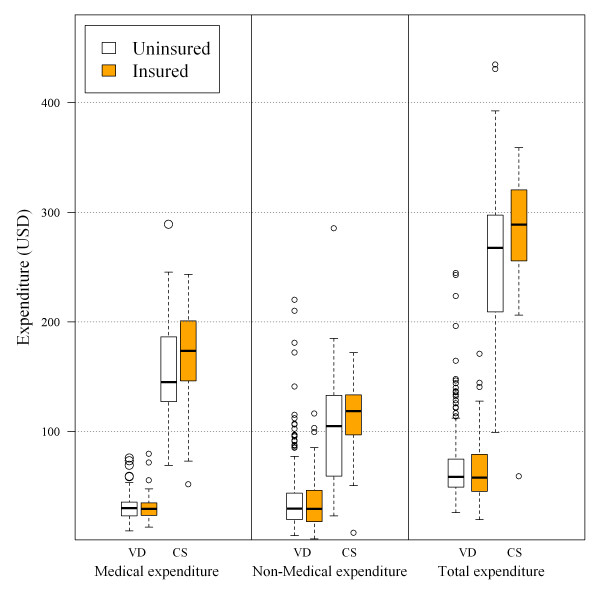
**Comparison of delivery care expenses stratified by type of expenditure, mode of delivery and availability of health insurance**. Lower and upper border of box: 1st quartile and 3rd quartile. The lines inside the box represent median of delivery care expenditure. The coloured boxes represent health insurance coverage. VD is vaginal delivery and CS is caesarean section.

Details of total health care expenses by mode of delivery are presented in Table 2. Both medical and non-medical care expenses for caesarean section were significantly higher than for vaginal delivery. The average total expenditure for vaginal delivery was found to be 59 USD (range: 48-75) and for caesarean section 270 USD (range: 218-312). Figure 1 shows a comparison of delivery care expenditure among the two modes of delivery and availability of health insurance. There was a significant difference in all types of expenditure by mode of delivery; however, no significant differences of expenditures were detected between the insured and the uninsured.

**Table 2:** Comparison of health care expenditure by mode of delivery

Expenditure	Mode of delivery		*P*-value
		
	Vaginal delivery	Caesarean section	
**Medical expenses**	30 (23, 35)	154 (129, 190)	< 0.001
**Non-medical expenses**	29 (19, 44)	111 (70, 133)	< 0.001
Transportation	3 (1, 10)	5 (3, 10)	0.007
Food	6 (5, 10)	30 (20, 40)	< 0.001
Additional costs	10 (0, 20)	50 (20, 60)	< 0.001
Productivity loss	3 (2, 7)	13 (6, 27)	< 0.001
**Total expenditure**	59 (48, 75)	270 (218, 312)	< 0.001

Numbers shown are mean expenses incurred in USD. Numbers in brackets are inter-quartile ranges

**Figure 2:**
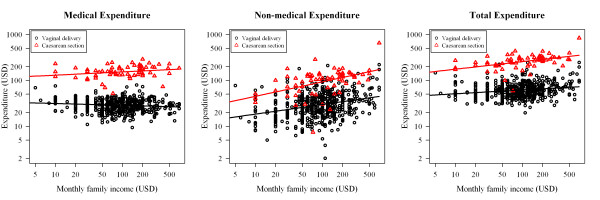
**Relationship between delivery care expenditure and monthly family income stratified by mode of delivery**. Dashed line: predicted line in caesarean section; solid line: predicted line in vaginal delivery.

Figure 2 shows the relationship between delivery care expenditure and monthly family income stratified by mode of delivery. Both axes are shown in logarithmic scales to facilitate interpretation. A significant interaction was found between family income and mode of delivery. For caesarean section only, higher family incomes were associated with higher medical expenses. For non-medical and total expenditures, higher family incomes were associated with higher expenses for both modes of delivery.

Factors associated with delivery care expenditure stratified by mode of delivery and adjusted for education are shown in Table 3. For caesarean section, medical expenses were higher for women with higher income; an increase in family income of 1 USD increased medical expenses by 0.09 USD on average. For non-medical and total expenditures, women with higher family income also had higher expenditures, more so for caesarean section; an increase in family income of 1 USD increased non-medical expenses by 0.07 USD and 0.39 USD on average for vaginal delivery and caesarean section, respectively. These results confirm the plots shown in Figure 2.

**Table 3:** Factors associated with delivery care expenditure stratified by mode of delivery

	Vaginal Delivery		Caesarean Section	
	**Coef**.	**95% CI**	**Coef**.	**95% CI**

***Medical expenditure***				
Family income^+^	-	-	0.09	0.02, 0.16
Hospital B*	10.35	8.98, 11.71	43.95	24.3, 63.6
***Non-medical expenditure***				
Family income^+^	0.07	0.05, 0.08	0.39	0.27, 0.51
Hospital B*	-6.79	-10.97, -2.62	-	-
***Total expenditure***				
Family income^+^	0.06	0.04, 0.08	0.43	0.28, 0.58
Hospital B*	-	-	-	-

Coef.: Coefficient. CI: 95% Confidence Interval

* Names of hospitals are not shown to protect confidentiality

^+ ^Monthly income in USD

Results are adjusted for education

### Effect of health insurance on delivery care services

Of all interviewed women, 25% of them were insured. Three-fourths of all women knew that health insurance was available and thought they had a right to health insurance. If they had health insurance, it would influence their access to more care. For women who were insured, 138 (95.8%) confirmed that the availability of health insurance affected their decision to receive delivery care in hospital because their out-of-pocket expenditures were subsidized for the medical care costs. Six women (4.2%) preferred delivery care in a hospital regardless of health insurance status.

For health providers, all SBAs and the directors of the two study hospitals agreed that the availability of health insurance influences the number of women accessing hospital services, especially delivery care. The directors said that health insurance has a positive effect on increasing hospital service utilization, in particular, for people who have low incomes. In addition, the availability of health insurance has also directly and indirectly affected the health providers and health facilities since the hospitals have to be responsible for some imbalance of health expenditures and revenue provided by the health insurance system.

## Discussion

Mode of delivery was associated with both medical and non-medical care expenditure after adjusting for family income, education and the hospital where delivery care was given. The effect of family income and hospital on all expenditures was significantly modified by mode of delivery. An increasing trend for non-medical care expenditure was observed in women with higher monthly family incomes for all women, while for medical expenditure, only for caesarean section. Health insurance was found to have no effect on all types of expenditure. One-fourth of women had health insurance and of these, all knew that they could use it for their delivery care utilization.

Significantly higher delivery care expenses for caesarean section were found in this study. From a literature search, there was one systematic review of economic aspects of alternative modes of delivery published in 2001 [18] and four separate studies published between 2002 and 2010 estimating the economic costs of delivery care [7-9,17]. All studies confirmed the high costs of delivery care for women undergoing caesarean section, although the methods of cost estimation varied. The studies included in the systematic review were conducted in developed countries and published before July 1999. Economic evaluation of alternative modes of delivery was not considered.

This study considered both medical and non-medical costs in the estimation of total delivery care expenditure for hospital-based services. A similar approach was used by the authors of two studies conducted in Nepal and Pakistan [7,17]. The average expenses incurred by each mode of delivery between these two countries were quite similar; however the studies were conducted 5 years apart. A study conducted in Scotland in 2002 considered only hospital costs and the findings confirmed that the cost for caesarean section was higher than for vaginal delivery [9]. Apart from different modes of delivery affecting costs, near-miss obstetric complications were also shown to increase the cost of care in vaginal delivery [8]. The reasons for higher costs between modes of delivery were due to medication costs and the length of stay in the hospital which influenced the additional costs for food, transportation and productivity loss [7,8,17]. However, it is not possible to make a direct comparison between modes of delivery or among countries in the cost of delivery care incurred due to the differences in estimation of delivery costs and inflation.

Family income, mode of delivery, and hospital were associated with medical and non-medical care expenditure in our study. Factors associated with health care expenditure were reported in one previous study in Pakistan which confirmed the effects of length of hospital stay and household income [7]. For the effect of hospital on medical expenditure, differences in the number of cases, length of hospital stay and room facilities may have contributed to this result, while for non-medical expenditure, the effect may have been due to differences in the socio-economic status of women. To our knowledge there has been no study identifying factors associated with medical and non-medical care expenditure altogether or investigating the effect of health insurance on delivery care expenditure.

Health insurance is a strategy of health financing reform to improve service utilization. Previous studies have confirmed that a positive impact on utilization of maternal health care services was due to health insurance [10,11]. A study in China reported lower total costs for delivery services among women who were insured [19]. In addition, a lower payment for insured women reduced the number of households that experienced catastrophic health expenditures [10]. In our study, only one-quarter of women were insured. The effects of health insurance on service utilization among this group were measured by direct interview. We did not analyze the deduction of medical care expenses covered by health insurance and the balance of hospital revenue.

This study had some limitations. First, the study was conducted in only two out of the 17 provincial hospitals in Lao PDR where emergency obstetric care and health insurance are available. Thus, generalisations of results from this study to the whole country need to be made with caution. Second, only health care expenses for delivery care services were measured. Third, medical costs incurred were from the hospital charges for the health care services.

Delivery at a facility by a skilled birth attendant is recommended to improve both maternal morbidity and mortality [20]; however, this delivery care results in an expense which contributes to household health care expenditure, especially for people who pay out-of pocket [3,4]. Knowing the cost of health care payment for hospital-based delivery care is crucial for women and their families in order to make financial preparations and in case out-of-pocket expenses are incurred. Our delivery care expenditure model can be used to calculate basic health financing by national policy makers to improve utilization of delivery care in hospitals.

## Conclusions

Substantially higher expenditure for hospital delivery care was detected for caesarean section compared to vaginal delivery. Three-fourths of the women who were not insured needed to be responsible for their own health care payment. Women with a higher family income were able to pay for more non-medical care expenses. The effect of health insurance on service utilization was noted by women and SBAs. To achieve the goal of utilizing delivery care in hospitals, coverage of health insurance needs to be expanded.

## Competing interests

The authors declare that they have no competing interests.

## Authors' contributions

DD designed this study and prepared the research protocol, field management and coordination, data collection and statistical analysis and a draft of the manuscript. TL provided supervision during the entire study and was involved during data collection, statistical analysis and manuscript preparation. EM was involved in statistical analysis and proof-reading of the final manuscript. All authors read and approved the final manuscript.
